# Annotations

**Published:** 1894-10-20

**Authors:** 


### Annotations.

### Mr. Bryant on the Public and the Profession.

Mr. Thomas Bryant delivered the opening lecture of the session to the members of the Hunterian Society last week, and discussed with them some of the burning medical questions of the hour. It will be admitted that the profession is at once restless and apathetic; willing to go forward, but afraid to move. Mr. Bryant does not sympathise with such a combination of strength and weakness. He told the members of the Hunterian Society that the profession was in the habit of going "cap in hand" to the two Royal colleges, and asking them to protect it against competition within its own ranks, medical aid associations, bogus private dispensaries, prescribing chemists, quacks, and all the other "animal parasites" which prey upon and half ruin the body corporate of physic. It is of no use going to the colleges, said Mr. Bryant, for much as they wish to help, they have not the power. "Why do the members of the profession not help themselves?" was the burden of his song. Medical men cannot remain *in statu pupillarii* throughout their lives any more than any other class. When they have left hospital and college and taken up practice on their own account, they must act the part of independent persons and fight their own battles. You have your voluntary associations, he said, your British Medical Association, your Defence Unions, your Protection Societies, and what not—why do you not put some life into them; make them powerful; give them authority to deal with the legislature on your behalf, and with the public generally? This is your line; rouse yourselves, be men and not children. It is those who know what they want, who want only right things, and who unceasingly insist upon having them who finally gain their ends.

### Sir James Paget on the Scientific Temper.

The medical profession loves Sir James Paget, and pays great heed to his *ex-cathedra* utterances. Some things which he said at the opening of the winter session of the Abernethian Society at St. Bartholomew's Hospital last week are of more than usual significance to the working medical practitioner at the present time. Practitioners of the "rank and file" are apt to feel somewhat depressed as regards their influence on science compared with those who love to call themselves "specialists" and "scientists" *par excellence*. Sir James Paget assures us that it is the truly practical man who is really scientific; that, indeed, there cannot be a divorce between science and practice. For, if we understand his contention aright, he holds that practice which is not scientific is mere quackery. Sir James insists that one thing above all others is essential to scientific practice, and that is the "scientific temper." This, he contends, is infinitely more important than the mere possession of knowledge. The man who "knows" is not always scientific in temper; the man who is scientific in temper always "knows," sooner or later, for his temper leaves him no contentment of mind until he arrives at knowledge. "There are certain parts of medicine which can be better studied in private practice than at a great hospital," says Sir James Paget. This pronouncement ought to go through and through the profession, and should be made clear to the public mind as well. The tendency of late years has been to hold that the mere general practitioner could be nothing more than a routine workman at the art of physic. Not so, argues the Nestor of English medicine. The family practitioner has departments of his own in which he can advance the pure science of medicine. Two lines of inquiry were specially mentioned in this connection. The first is the investigation of mixed diseases, and the second the influence of medicines on the body. In other words, in the practical and all-important region of general therapeutics, Sir James Paget holds that the family practitioner has better opportunities of advancing the science and art of medicine than the specialist or the hospital physician. This will be found a welcome assurance to the family doctor; and we sincerely hope he will pluck up heart, and take his proper place among the scientific students of the times.

### The Clinical Society.

At the meeting of the Clinical Society, on October 12th, Dr. Soltau Fenwick drew attention to a fatal form of tetany which occasionally occurs in the course of dilatation of the stomach. It may not happen very often, but a considerable proportion of the cases in which it does occur end fatally. It is very important, therefore, to beware of the meaning and gravity of tetany when it is found complicating such a condition as dilatation of the stomach, for although at first the symptoms may seem insignificant, and to those not aware of it, may appear to have no special relationship with the disease in question, they really mark the onset of an urgent complication, liable to bring to an unexpected end a case previously looked upon as of quite a chronic nature and free from all immediate risk. Sometimes the spasms are confined to the hands and feet—these are the less fatal cases; where, however, they extend to the face, the neck, and the muscles of respiration, and especially when they are complicated with epileptiform seizures, they are of extremely grave omen. In some cases they appear to be rapidly fatal, but in others recur at considerable intervals, the attacks gradually becoming more and more severe, and involving fresh areas, until the spasms reach the respiratory muscles and end in death. As to ætiology, it is thought probable that the attacks are due to some toxic product of fermentation in the stomach, and it is noteworthy that in a case mentioned by Dr. Fenwick, after many paroxysms had taken place, they ceased completely and permanently after careful treatment by stomach washing. To some patients no doubt the use of the tube seems a revolting process, and they prefer to put up with the troubles of their malady rather than submit to the inconvenience of its cure. The physician may advise, but can hardly insist. What, however, is worth remembering is that the whole aspect of the case is changed by the onset of tetany; instead of a chronic affair, the condition becomes one of definite gravity, and the reasonable treatment which the physician may fairly insist upon is the careful lavage of the stomach, and the adoption of means to empty, and perhaps to disinfect, the intestinal canal.

